# Supplementary material for: Rapid Identification of Constituents in Polygonatum cyrtonema Hua Using UHPLC-Q-Exactive Orbitrap Mass Spectrometry
Source: Molecules. 2025 Feb 5;30(3):723. doi: 10.3390/molecules30030723 (PMC11819922; doi:10.3390/molecules30030723)
Supplement: Supplementary file 1 [file molecules-30-00723-s001.zip › molecules-3398127-supplementary.pdf]

Supplementary Table S1: Retention times and mass spectral data of PCH.

Peak	RT (min)	Theoretical Mass m/z	Experimental Mass m/z	Error (ppm)	Formula	MS/MS fragment (+)	MS/MS fragment (-)	Identification	A	W	T	S	F	E	N
1**	0.68	175.1190	175.1187	-1.4	$C_6H_{14}N_4O_2$	MS^2[175]: 116.0707(100), 70.0657(43), 158.0922(19), 130.0974(18)		argininic acid	+	+	+	+	+	+	+
2**	0.81	112.0505	112.0506	0.6	$C_4H_5N_3O$	MS^2[112]: 95.0242(100), 69.0453(38), 94.0402(10)		cytosine	+	+	+	+	+	+	+
3**	0.83	127.0389	127.0388	-1.3	$C_6H_6O_3$	MS^2[127]: 108.0445(100), 109.0285(60), 81.0339(28), 53.0392(12)		5-hydroxymethylfurfural	+	+	+	+	+	+	+
4**	0.83	116.0706	116.0707	1.2	$C_5H_9NO_2$	MS^2[116]: 70.0656(100), 98.0604(2), 88.0396(2)		proline	+	+	+	+	+	+	+
5**	0.90	268.1040	268.1032	-2.9	$C_{10}H_{13}N_5O_4$	MS^2[268]: 136.0615(100), 57.0343(1)		adenosine	-	+	+	+	+	+	+
6**	0.91	191.0197	191.0188	-4.7	$C_6H_8O_7$		MS^2[191]: 87.0074(100), 111.0074(62), 85.0280(22)	citric acid	-	+	+	+	+	+	-
7**	0.91	130.0863	130.0861	-1.1	$C_6H_{11}NO_2$	MS^2[130]: 84.0812(100), 56.0501(2)		pipercoic acid	+	+	+	+	+	+	+
8**	0.91	166.0863	166.0860	-1.1	$C_9H_{11}NO_2$	MS^2[166]: 123.0440(100) 120.0808(9), 103.0544(8), 149.0245(5)		phenylalanine	+	+	+	+	+	+	+
9**	0.91	180.0666	180.0658	-4.5	$C_9H_{11}NO_3$		MS^2[180]: 163.0390(100), 119.0489(47), 72.0077(25)	L-tyrosine	+	+	+	+	+	-	-
10*	0.92	133.0142	133.0140	-1.9	$C_4H_6O_5$		MS^2[133]: 115.0024(100), 71.0124(42),	L-(-)-Malic acid	+	+	+	+	+	-	-

							89.0230(7)								
11***	1.27	130.0863	130.0860	-1.7	$C_6H_{11}NO_2$	MS^2[130]: 84.0812(100), 56.0503(2)		pipercoic acid isomer	-	-	+	+	+	+	+
12***	1.29	127.0389	127.0387	-1.8	$C_6H_6O_3$	MS^2[127]: 109.0285(100), 81.0339(32), 53.0393(9)		5-hydroxymethylf urfural isomer	+	+	+	+	+	+	+
13***	1.29	166.0863	166.0859	-1.7	$C_9H_{11}NO_2$	MS^2[166]: 123.0440(100) 120.0805(2), 103.0544(3), 149.0245(5)		phenylalanine isomer	+	+	+	+	+	+	+
14**	1.30	191.0197	191.0188	-4.7	$C_6H_8O_7$		MS^2[191]: 109.0282(100), 111.0074(56), 85.0280(22), 87.0073(21)	citric acid	-	+	+	+	+	+	-
15**	1.30	130.0499	130.0497	-1.1	$C_5H_7NO_3$	MS^2[130]: 84.0812(100), 70.0657(22), 56.0502(2)		L-pyroglutamicac id	+	+	+	+	+	+	+
16**	1.31	180.0666	180.0658	-4.5	$C_9H_{11}NO_3$		MS^2[180]: 163.0389(100), 119.0489(54), 72.0076(27)	L-tyrosine	+	+	+	+	+	-	-
17**	1.36	268.1040	268.1033	-2.6	$C_{10}H_{13}N_5O_4$	MS^2[268]: 136.0615(100), 57.0342(4),		adenosine	-	+	+	+	+	+	+
18**	1.43	248.1492	248.1488	-1.9	$C_{11}H_{21}NO_5$	MS^2[248]: 230.1383(100), 194.1185(8), 212.1284(2)		pantothenic acid	+	+	+	+	+	+	+
19**	1.48	284.0989	284.0981	-2.8	$C_{10}H_{13}N_5O_5$	MS^2[284]: 152.0564(100), 85.0287(2), 135.0303(2)		isoguanosine	+	+	+	+	+	-	-
20***	1.54	248.1492	248.1488	-1.9	$C_{11}H_{21}NO_5$	MS^2[248]: 230.1383(100), 194.1185(1), 212.1279(3)		pantothenic acid isomer	+	+	+	+	+	-	-
21***	2.32	127.0389	127.0388	-1.3	$C_6H_6O_3$	MS^2[127]: 109.0285(100),		5-hydroxymethylf urfural isomer	+	-	+	+	-	-	-

						81.0340(32), 53.0393(8)									
22***	2.53	166.0863	166.0860	-1.1	$C_9H_{11}NO_2$	MS^2[166]: 123.0440(100) 120.0808(5), 103.0545(3), 149.0245(2)		phenylalanine isomer	+	+	+	+	+	+	+
23***	3.51	248.1492	248.1487	-2.1	$C_{11}H_{21}NO_5$	MS^2[248]: 230.1382(100), 212.1284(7), 194.1185(1),		pantothenic acid isomer	+	-	+	+	+	+	+
24*	3.73	153.0193	153.0186	-4.6	$C_7H_6O_4$		MS^2[153]: 109.0281(100), 81.0332(3), 125.0233(3)	3,4-dihydroxybenzoic acid	+	+	+	+	+	-	-
25*	4.05	205.09715	205.0967	-2.0	$C_{11}H_{12}N_2O_2$	MS^2[205]: 188.0703(100), 146.0597(51), 159.0913(7), 118.0651(3)		L-tryptophan	+	+	+	+	+	+	+
26*	4.25	353.0878	353.0879	0.2	$C_{16}H_{18}O_9$		MS^2[353]: 135.0442(100), 191.0555(82), 179.0342(27)	neochlorogenic acid	-	-	+	+	-	-	-
27*	4.80	330.1699	330.1693	-2.0	$C_{19}H_{23}NO_4$	MS^2[330]: 239.0697(100), 255.1009(54), 181.0642(9), 123.0802(2)		Sinomenine	+	+	+	+	+	+	+
28***	4.93	137.0244	137.0240	-4.7	$C_7H_6O_3$		MS^2[137]: 137.0232(100), 93.0332(23)	Salicylic acid isomer	-	-	-	+	+	-	-
29***	5.10	137.0244	137.0241	-4.3	$C_7H_6O_3$		MS^2[137]: 93.0332(100), 137.0233(28)	Salicylic acid isomer	-	-	+	+	-	-	-
30**	5.17	215.0826	215.0820	-2.7	$C_{12}H_{12}N_2O_2$		MS^2[215]: 171.0917(100), 116.0493(72), 142.0650(24)	2,3,4,6-tetrahydro-1H-β-carboline-3-carboxylic acid	+	-	-	+	+	-	-
31**	5.17	341.0878	341.0880	0.6	$C_{15}H_{18}O_9$		MS^2[341]: 161.0597(100), 179.0347(10), 101.0237(6), 135.0449(2)	caffeic acid-hexoside	+	-	-	+	+	-	-

32***	5.22	205.0972	205.0970	-0.8	$C_{11}H_{12}N_2O_2$	MS^2[205]: 117.0702(100), 118.0652(62), 146.0598(45), 188.0704(2)		L-tryptophan isomer	+	-	+	+	+	+	+
33***	5.22	248.1492	248.1488	-1.9	$C_{11}H_{21}NO_5$	MS^2[248]: 230.1382(100), 212.1275(7), 194.1185(1)		pantothenic acid isomer	+	-	+	+	+	+	-
34*	5.24	353.0878	353.0877	-0.3	$C_{16}H_{18}O_9$		MS^2[353]: 191.0551(100), 179.0341(2), 161.0233(2)	chlorogenic acid	-	-	+	+	-	-	-
35*	5.32	353.0878	353.0885	1.9	$C_{16}H_{18}O_9$		MS^2[353]: 191.0551(100), 179.0339(12), 161.0230(2) 135.0441(2)	cryptochlorogenic acid	-	-	+	+	-	-	-
36**	5.32	490.2072	490.2064	-1.6	$C_{25}H_{31}NO_9$	MS^2[490]: 328.1536(100), 265.0853(17), 297.1108(9)		11-glc-norisocory dine	-	-	-	+	+	+	-
37***	5.35	215.0826	215.0818	-3.5	$C_{12}H_{12}N_2O_2$		MS^2[215]: 171.0917(100), 116.0494(86), 142.0657(34)	2,3,4,6-tetrahydro -1H-β-carboline-3 -carboxylic acid isomer	+	-	-	+	+	-	-
38**	5.41	285.1245	285.12454	0.3	$C_{16}H_{18}N_2O_3$		MS^2[285]: 167.0605(100), 207.0920(14), 195.0553(11)	5(9H-β-carbolin-1-yl)-pentane-1,2, 5-triol	+	-	-	+	+	+	+
39**	5.50	328.1549	328.1535	-2.5	$C_{19}H_{22}NO_4^+$	MS^2[328]: 89.0600(100), 265.0853(36), 58.0658(25), 283.0956(4)		boldine	-	+	+	+	+	-	-
40**	5.50	344.1862	344.1851	-1.7	$C_{20}H_{26}NO_4^+$	MS^2[344]: 175.0751(100), 299.1275(89), 137.0594(71), 143.0491(24)		zizyphusine+2H	-	-	+	+	+	+	+
41*	5.65	179.0349	179.0342	-4.6	$C_9H_8O_4$		MS^2[179]: 135.0439(100), 179.0339(49), 164.0105(26)	caffeic acid	+	-	+	+	-	-	-

42***	5.67	328.1549	328.1538	-3.3	$C_{19}H_{22}NO_4^+$	MS^2[328]: 283.0956(100), 58.0658(10), 89.0600(5), 265.0850(4)		boldine isomer	-	+	+	+	+	-	-
43*	5.83	375.1310	375.1290	-5.0	$C_{17}H_{20}N_4O_6$		MS^2[375]: 212.0823(100), 255.0885(64), 241.0726(37)	riboflavin	-	-	+	+	-	-	-
44***	5.95	328.1549	328.1535	-2.5	$C_{19}H_{22}NO_4^+$	MS^2[328]: 265.0854(100), 58.0659(10), 283.0956(4)		boldine isomer	-	+	+	+	+	+	+
45**	5.95	358.2018	358.2006	-1.9	$C_{21}H_{28}NO_4^+$	MS^2[358]: 192.1015(100), 58.0658(18), 281.1143(2), 313.1413(6)		pareirarinea	-	-	-	+	+	+	-
46***	6.05	328.1549	328.1538	-3.3	$C_{19}H_{22}NO_4^+$	MS^2[328]: 265.0852(100), 58.0658(10), 283.0955(8), 89.0600(5)		boldine isomer	-	+	+	+	+	+	+
47***	6.08	374.1604	374.1591	-1.9	$C_{20}H_{24}NO_6^+$	MS^2[374]: 329.1013(100), 58.0658(3), 297.1104(3)		di-hydroxylation of magnoflorine isomer	-	-	-	+	-	+	+
48**	6.10	342.1705	342.1688	-5.0	$C_{20}H_{24}NO_4^+$	MS^2[342]: 192.1014(100), 151.0753(2), 177.0783(2)		phellodendrine	+	+	+	+	+	+	+
49**	6.25	356.1862	356.1847	-2.7	$C_{21}H_{26}NO_4^+$	MS^2[356]: 58.0658(100), 279.1009(99), 311.1271(54) 280.1075(26), 296.1038(16), 251.1072(9)		menisperine	+	+	+	+	+	+	+
50**	6.36	200.0706	200.0702	-2.0	$C_{12}H_9NO_2$	MS^2[200]: 172.0753(100), 182.0597(31), 157.0523(5)		dictamnine	+	-	+	+	+	+	+
51**	6.44	433.1140	433.1148	1.8	$C_{21}H_{22}O_{10}$		MS^2[433]: 271.0610(100),	Naringenin 7-O-glucoside	-	-	-	-	+	-	-

							119.0491(36), 151.0028(30)								
52**	6.45	193.0506	193.0497	-4.6	$C_{10}H_{10}O_4$		MS^2[193]: 149.0596(100), 178.0262(11), 134.0361(2)	ferulic acid	+	-	+	+	-	-	-
53*	6.45	609.1461	609.1460	-0.2	$C_{27}H_{30}O_{16}$		MS^2[609]: 300.0274(100), 301.0346(51), 151.0024(4)	rutin	+	-	-	+	+	+	+
54**	6.46	298.1085	298.1082	-0.9	$C_{17}H_{17}NO_4$		MS^2[298]: 280.0978(100), 119.0490(34), 145.0284(33), 160.0394(13), 134.0600(3), 117.0335(2)	coumaroyloctopamine	+	-	-	+	+	+	+
55*	6.54	163.0401	163.0392	0.2	$C_9H_8O_3$		MS^2[163]: 119.0489(100), 163.0389(22), 120.0522(5)	p-Coumaric acid	+	-	+	+	+	+	-
56***	6.55	374.1604	374.1590	-2.2	$C_{20}H_{24}NO_6^+$	MS^2[374]: 329.1012(100), 58.0658(4), 297.0763(1)		di-hydroxylation of magnoflorine isomer	-	-	-	+	+	+	+
57*	6.56	593.1512	593.1519	1.2	$C_{27}H_{30}O_{15}$		MS^2[593]: 284.0325(100), 178.9984(2), 151.0030(2)	Kaempferol 3-glucorhamnoside	-	+	+	+	+	+	+
58*	6.57	173.0819	173.0812	-4.5	$C_8H_{14}O_4$		MS^2[173]: 111.0802(100), 129.0908(5), 155.0701(2)	octanedioic acid	+	+	+	+	+	+	+
59***	6.58	179.0349	179.0342	-4.1	$C_9H_8O_4$		MS^2[179]: 179.0338(30), 164.0103(50), 135.0442(7)	caffeic acid isomer	-	-	-	-	+	-	-
60**	6.58	296.1651	296.1637	-2.8	$C_{19}H_{22}NO_2^+$	MS^2[296]: 251.1060(100), 58.0658(20), 219.0799(14), 221.0964(2), 236.0829(2)		C_1-demethoxy-C_2-dehydroxof magnoflorine	+	+	+	+	-	+	+

61**	6.64	1093.5072	1093.5054	-1.7	$C_{51}H_{82}O_{25}$		MS^2[1093]:<br>931.4555(100),<br>769.4042(79),<br>751.3934(68)	kingianoside E	+	-	-	+	+	+	+
62**	6.65	282.1489	282.1482	-2.4	$C_{18}H_{19}NO_2$	MS^2[282]:<br>250.0984(100),<br>234.1035(35),<br>237.0903(2)		N-nornuciferine	-	-	+	+	+	+	+
63**	6.66	317.0655	317.0648	-2.6	$C_{16}H_{12}O_7$	MS^2[317]:<br>302.0415(100),<br>285.0389(43),<br>257.0444(12)		Isorhamnetin	-	-	-	+	+	-	-
64*	6.67	463.0882	463.0884	0.4	$C_{21}H_{20}O_{12}$		MS^2[463]:<br>300.0277(100),<br>271.0251(25),<br>255.0301(12),<br>151.0029(10)	hyperoside	-	-	-	+	+	-	-
65**	6.72	328.1190	328.1189	-0.4	$C_{18}H_{19}NO_5$		MS^2[328]:<br>310.1085(100),<br>161.0233(71),<br>175.0390(12),<br>135.0441(8),<br>160.0395(4),<br>134.0362(2)	N-feruloyloctopa<br>mine	+	-	-	+	+	+	+
66***	6.72	356.1862	356.1845	-3.1	$C_{21}H_{26}NO_4^+$	MS^2[356]:<br>58.0658(100),<br>296.1038(16),<br>251.1072(9),<br>279.1004(4),<br>311.1279(2),<br>280.1080(2)		menisperine<br>isomer	-	+	+	+	+	+	+
67*	6.72	447.0933	447.09323	-0.1	$C_{21}H_{20}O_{11}$		MS^2[447]:<br>284.0326(100),<br>285.0390(64)	Luteolin-7-O-glu<br>coside	-	+	-	+	+	-	-
68**	6.73	177.0546	177.05428	-3.7	$C_{10}H_8O_3$	MS^2[177]:<br>145.0281(100),<br>177.0543(47),<br>149.0594(12),<br>121.0648(3)		4-Methylumbellif<br>erone	+	-	-	+	+	+	+
69***	6.75	298.1085	298.1083	-0.8	$C_{17}H_{17}NO_4$		MS^2[298]:<br>280.0978(100),<br>119.0490(43),<br>145.0284(45),<br>160.0394(14),	coumaroyloctopa<br>mine isomer	+	-	-	+	+	+	+

							134.0600(4), 117.0334(2)								
70**	6.75	370.1649	370.1643	-1.7	$C_{21}H_{23}NO_5$	MS^2[370]: 58.0658(100), 293.0803(87), 265.0858(8), 137.0591(4)		allocryptopine	-	-	-	+	+	+	+
71**	6.77	163.0401	163.0392	0.2	$C_9H_8O_3$		MS^2[163]: 119.0489(100), 120.0527(4)	p-Coumaric acid	-	-	+	-	-	-	-
72**	6.78	294.1489	294.14825	-2.1	$C_{19}H_{19}NO_2$	MS^2[294]: 217.0643(100), 279.1247(10), 250.0938(7), 263.1287(3), 58.0656(1)		dehydronuciferine	-	-	+	+	+	+	+
73***	6.78	1093.5072	1093.5071	-0.1	$C_{51}H_{82}O_{25}$		MS^2[1093]: 931.4555(100), 769.4042(79), 751.3934(68)	kingianoside E isomer	+	-	-	+	+	+	+
74*	6.87	593.1512	593.1505	-1.2	$C_{27}H_{30}O_{15}$		MS^2[593]: 285.0399(100), 284.0323(91), 255.0296(26), 227.0340(9), 151.0022(5)	nicotiflorin	-	-	-	+	+	-	-
75*	6.91	193.0506	193.0497	-4.6	$C_{10}H_{10}O_4$		MS^2[193]: 178.0260(100), 149.0597(14), 134.0361(13)	ferulic acid	+	-	-	+	+	-	-
76**	6.92	317.0655	317.0649	-2.1	$C_{16}H_{12}O_7$	MS^2[317]: 302.0417(100), 285.0385(39), 257.0444(17)		isorhamnetin	-	-	-	+	+	-	-
77**	6.92	623.1618	623.1604	-2.2	$C_{28}H_{32}O_{16}$		MS^2[623]: 315.0509(100), 300.0273(6), 151.0023(2), 271.0258(2)	methyl-quercetin-O-rutinoside	-	-	-	+	+	+	+
78*	6.94	303.0510	303.0508	-0.6	$C_{15}H_{12}O_7$		MS^2[303]: 125.0230(100), 285.0401(51), 175.0390(21)	taxifolin	-	-	-	-	+	+	-

79**	6.95	931.4544	931.4545	0.1	$C_{45}H_{72}O_{20}$		MS^2[931]: 751.3914(100), 769.4042(44), 607.5002(8)	kingianoside C	+	-	-	+	+	+	+
80***	7.00	177.0546	177.0541	-3.1	$C_{10}H_8O_3$	MS^2[177]: 145.0282(100), 177.0542(54), 149.05945(20), 121.0648(2)		4-Methylumbelliferone isomer	+	-	-	+	+	+	+
81***	7.00	328.1190	328.1188	-0.6	$C_{18}H_{19}NO_5$		MS^2[328]: 310.1086(100), 161.0234(98), 175.0392(13), 135.0439(10), 160.0397(6), 134.0365(5),	N-feruloyloctopamine isomer	+	-	-	+	+	+	+
82***	7.03	374.1604	374.1590	-2.2	$C_{20}H_{24}NO_6^+$	MS^2[374]: 329.1011(100), 58.0658(3), 297.1104(3)		di-hydroxylation of magnoflorine isomer	-	-	-	-	+	+	+
83**	7.05	356.1498	356.1487	-1.4	$C_{20}H_{22}NO_5^+$	MS^2[356]: 58.0658(100), 311.1269(22), 279.1006(7), 251.1061(1)		C_5-methylene to ketone of magnoflorine	-	-	-	-	-	+	+
84*	7.07	447.0933	447.0936	0.7	$C_{21}H_{20}O_{11}$		MS^2[447]: 284.0329(100), 255.0300(67), 285.0395(30)	astragalin	-	-	-	-	+	+	+
85**	7.08	344.1498	344.1488	-1.4	$C_{19}H_{22}NO_5^+$	MS^2[344]: 283.0959(100), 58.0659(9), 265.0852(3)		N-CH_3-hydroxylation of C_2-O-demethylation of magnoflorine	-	-	+	+	+	+	+
86*	7.11	193.0506	193.0497	-4.6	$C_{10}H_{10}O_4$		MS^2[193]: 61.9870(100), 133.0282(3), 134.0367(2)	isoferulic acid	-	-	-	+	+	-	-
87*	7.14	282.1489	282.1485	-1.4	$C_{18}H_{19}NO_2$	MS^2[282]: 250.0984(100), 234.1035(35), 237.0908(5)		N-nornuciferine	-	-	+	+	+	+	+
88**	7.17	303.0863	303.0855	-2.8	$C_{16}H_{14}O_6$	MS^2[303]: 123.0439(100),		hematoxylin	-	+	+	-	+	-	-

						131.0489(84), 149.0596(49)									
89*	7.17	609.1825	609.1822	-0.5	$C_{28}H_{34}O_{15}$		MS^2[609]: 301.0717(100), 302.0745(6), 125.0230(2)	hesperidin	-	+	+	+	+	+	+
90***	7.19	294.1489	294.1480	-2.8	$C_{19}H_{19}NO_2$	MS^2[294]: 58.0658(100), 279.1244(3), 217.0643(2), 250.0940(2)		dehydronuciferin e isomer	-	-	+	+	+	+	+
91**	7.19	308.1281	308.1272	-3.0	$C_{19}H_{17}NO_3$	MS^2[308]: 162.0546(100), 249.0903(85), 219.0809(3)		N-acetylanonaine	-	-	-	+	+	+	+
92**	7.19	338.1392	338.1381	-3.4	$C_{20}H_{20}NO_4^+$	MS^2[338]: 323.1144(100), 322.1071(20), 294.1101(4)		columbamine	-	-	-	+	+	+	+
93*	7.20	280.1332	280.1330	-0.7	$C_{18}H_{17}NO_2$	MS^2[280]: 249.0911(100), 219.0806(24), 191.0856(11)		roemerine	-	-	+	+	+	+	+
94**	7.24	296.1651	296.1638	-2.5	$C_{19}H_{22}NO_2^+$	MS^2[296]: 251.1061(100), 58.0658(20), 219.0800(13)		C_1-demethoxy-C_2 -dehydrox of magnoflorine	-	-	-	-	-	+	+
95***	7.30	303.0863	303.0856	-2.5	$C_{16}H_{14}O_6$	MS^2[303]: 123.0440(100), 131.0491(3), 149.0600(3)		hematoxylin isomer	+	-	-	-	+	-	-
96*	7.30	431.0984	431.0993	2.2	$C_{21}H_{20}O_{10}$		MS^2[431]: 268.0379(100), 269.0454(49)	apigenin-7-glucos ide	-	-	-	-	+	-	-
97**	7.37	1237.5495	1237.5502	0.5	$C_{57}H_{90}O_{29}$		MS^2[1237]: 913.4460(100), 1075.4954(91), 751.3897(41), 571.3272(29)	kingianoside Z	-	-	-	-	+	+	-
98***	7.37	1241.5808	1241.5797	-0.9	$C_{57}H_{94}O_{29}$		MS^2[1241]: 755.5413(100), 1079.6985(92), 917.6189(88)	flavopiridol glycosides isomer	-	-	-	-	+	+	-

99***	7.38	303.0863	303.0859	-1.2	$C_{16}H_{14}O_6$	MS^2[303]: 123.0440(100), 131.0491(3), 149.0600(3)		hematoxylin isomer	+	-	-	-	+	-	-
100*	7.49*	435.1297	435.1286	-2.4	$C_{21}H_{24}O_{10}$		MS^2[435]: 167.0339(100), 273.0767(23), 179.0340(10)	phlorizin	-	-	-	+	+	+	+
101*	7.54	187.0975	187.0967	-4.9	$C_9H_{16}O_4$		MS^2[187]: 125.0959(100), 187.0967(67), 141.8669(18)	azelaic acid	+	+	+	+	+	+	+
102**	7.57	623.3116	623.31061	-1.5	$C_{38}H_{42}N_2O_6$	MS^2[623]: 250.0982(59), 251.1058(61)		tetrandrine	-	-	-	+	+	+	+
103***	7.63	303.0863	303.0855	-2.7	$C_{16}H_{14}O_6$	MS^2[303]: 123.0440(100), 149.0598(22), 131.0491(3)		hematoxylin isomer	-	-	-	-	+	+	+
104***	7.68	179.0349	179.0342	-4.5	$C_9H_8O_4$		MS^2[179]: 134.9868(100), 164.0109(20), 179.0341(17)	caffeic acid isomer	-	-	-	-	+	+	+
105***	7.81	163.0401	163.0393	-4.8	$C_9H_8O_3$		MS^2[163]: 162.8379(100), 119.0489(80), 120.0521(3)	p-Coumaric acid isomer	-	-	+	+	-	-	-
106**	7.84	1241.5808	1241.5819	0.9	$C_{57}H_{94}O_{29}$		MS^2[1241]: 755.5413(100), 917.6189(98), 1079.6985(61)	flavopiridol glycosides	-	-	-	-	+	+	-
107***	7.92	1237.5495	1237.5480	-1.2	$C_{57}H_{90}O_{29}$		MS^2[1237]: 913.4460(100), 1075.4954(70), 751.3897(43), 571.3272(24),	kingianoside Z isomer	-	-	-	-	+	+	-
108*	7.94	137.0244	137.0237	-4.7	$C_7H_6O_3$		MS^2[137]: 93.0332(100), 137.0232(52)	salicylic acid	+	-	+	+	+	-	-
109**	7.94	593.3684	593.3678	-1.0	$C_{33}H_{52}O_9$	MS^2[593]: 287.2007(100), 269.1902(72), 145.0493(8), 251.1790(5)		huangjinoside D	+	-	-	-	+	+	+

110**	8.12	1075.4966	1075.4954	-1.2	$C_{51}H_{80}O_{24}$		MS^2[1075]: 221.0660(100), 913.4478(31), 179.0552(6)	cyrtonemoside A	+	-	-	+	+	+	+
111***	8.13	177.0546	177.0541	-3.1	$C_{10}H_8O_3$	MS^2[177]: 145.0281(100), 177.0542(51), 149.05945(13), 121.0642(3)		4-Methylumbelliferone isomer	+	-	-	-	+	+	+
112**	8.13	312.1241	312.1243	-0.9	$C_{18}H_{19}NO_4$		MS^2[312]: 297.1005(100), 191.0580(3)	N-trans-Feruloyltyramine	+	-	-	+	+	+	+
113**	8.27	263.1288	263.1287	-0.5	$C_{15}H_{20}O_4$		MS^2[263]: 122.0362(100), 151.0752(32), 125.0595(31)	abscisic acid	+	-	+	+	+	+	-
114***	8.36	593.3684	593.3677	-1.1	$C_{33}H_{52}O_9$	MS^2[593]: 287.2007(100), 269.1902(10), 251.1794(2), 145.0500(2)		huangjinoside D isomer	+	+	+	+	+	+	+
115***	8.49	177.0546	177.0542	-2.2	$C_{10}H_8O_3$	MS^2[177]: 145.0280(11), 149.05945(75), 177.0542(62), 121.0647(43)		4-Methylumbelliferone isomer	+	-	-	-	+	+	+
116**	8.49	312.1241	312.1243	-0.9	$C_{18}H_{19}NO_4$		MS^2[312]: 297.1005(100), 191.0578(7)	N-trans-Feruloyltyramine	+	-	-	+	+	+	+
117**	8.52	1031.5421	1031.5372	-4.8	$C_{51}H_{82}O_{21}$	MS^2[1031]: 415.3232(100), 869.4947(97), 577.3772(14), 723.4373(10)		pseudoprotodioscin	-	-	-	-	+	-	-
118**	8.55	299.0924	299.0922	-1.1	$C_{17}H_{16}O_5$		MS^2[299]: 205.0501(100), 193.0502(93), 281.1767(10)	5,7-dihydroxy-8-methyl-3-(4-hydroxybenzyl)-chromate-4-one	-	-	-	-	+	+	+
119*	8.65	255.0663	255.0661	-0.7	$C_{15}H_{12}O_4$		MS^2[255]: 119.0490(100), 219.8448(45), 135.0076(37), 153.0182(17)	liquiritigenin	-	-	-	-	-	+	-

120*	8.79	263.1288	263.1287	-0.6	$C_{15}H_{20}O_4$		MS^2[263]: 122.0360(100), 125.0596(43), 151.0755(32)	abscisic acid	+	-	+	+	+	+	-
121***	8.84	205.0972	205.0971	-0.2	$C_{11}H_{12}N_2O_2$	MS^2[205]: 188.0702(100), 146.0597(48), 159.0913(6), 118.0651(3)		L-tryptophan isomer	-	-	-	+	+	+	+
122*	8.90	285.0405	285.0397	-2.6	$C_{15}H_{10}O_6$		MS^2[285]: 133.0285(100), 151.0029(14), 175.0401(9), 105.0336(5)	luteolin	-	-	-	-	-	+	+
123*	8.92	301.0354	301.0351	-0.9	$C_{15}H_{10}O_7$		MS^2[301]: 301.0349(100), 151.0024(93), 178.9976(51)	quercetin	-	-	-	+	+	+	+
124**	9.30	283.0612	283.0606	-2.3	$C_{16}H_{12}O_5$		MS^2[283]: 283.0612(100), 268.0372(35), 240.0421(12)	wogonin	-	-	-	-	+	+	+
125***	9.39	179.0349	179.0342	-4.5	$C_9H_8O_4$		MS^2[179]: 134.9866(100), 164.0104(40), 179.0341(28)	caffeic acid isomer	+	-	-	-	+	+	+
126***	9.67	193.0506	193.0498	-4.2	$C_{10}H_{10}O_4$		MS^2[193]: 178.0261(100), 149.0597(28), 134.0361(2)	ferulic acid isomer	+	-	-	+	+	+	-
127*	10.15	271.0612	271.0611	-0.7	$C_{15}H_{12}O_5$		MS^2[271]: 151.0027(100), 119.0491(33), 177.0181(13)	naringenin	+	-	-	-	+	+	+
128*	10.60	285.0405	285.0393	-4.2	$C_{15}H_{10}O_6$		MS^2[285]: 159.0439(100), 171.0447(70), 151.0029(49)	kaempferol	-	-	-	+	+	+	+
129**	10.67	235.1693	235.1687	-2.3	$C_{15}H_{22}O_2$	MS^2[235]: 199.1479(100), 189.1633(78), 157.1009(46)		curcumenol	+	-	-	-	+	+	+
130***	10.85	235.1693	235.1688	-2.2	$C_{15}H_{22}O_2$	MS^2[235]: 199.1479(100),		curcumenol isomer	+	-	-	-	+	+	+

						189.1637(67),<br>157.1009(33)									
131***	11.10	235.1693	235.1687	-2.3	$C_{15}H_{22}O_2$	MS^2[235]:<br>199.1476(100),<br>189.1632(92),<br>157.1005(31)		curcumenol isomer	+	-	-	-	+	+	+
132**	11.54	315.0874	315.0873	-0.5	$C_{17}H_{16}O_6$		MS^2[315]:<br>139.0389(100),<br>193.0497(44),<br>205.0500(10)	odoratumone B	+	+	+	+	+	+	+
133**	11.56	329.2333	329.2329	-1.2	$C_{18}H_{34}O_5$		MS^2[329]:<br>171.1018(100),<br>211.1334(68),<br>183.1379(7),<br>293.2126(7)	tianshic acid	+	+	+	+	+	+	+
134***	12.23	283.0612	283.0610	-0.5	$C_{16}H_{12}O_5$		MS^2[283]:<br>283.0612(100),<br>240.0421(25),<br>268.0375(6)	wogonin isomer	-	-	-	-	+	+	+
135*	12.34	267.0663	267.0669	2.3	$C_{16}H_{12}O_4$		MS^2[267]:<br>267.0659(100),<br>252.0423(83)	formononetin	-	-	-	-	+	+	+
136**	13.31	299.0924	299.0924	-0.2	$C_{17}H_{16}O_5$		MS^2[299]:<br>205.0501(100),<br>193.0502(93),<br>281.1767(10)	5,7-dihydroxy-8-methyl-3-(4-hydroxybenzyl)-chromate-4-one	+	+	+	+	+	+	+
137**	13.57	429.2999	429.2990	-2.3	$C_{27}H_{40}O_4$	MS^2[429]:<br>411.2886(100),<br>393.2779(86),<br>229.1593(9)		Neoruscogenin	+	+	+	+	+	+	+
138**	13.57	913.4439	913.4434	-0.5	$C_{45}H_{70}O_{19}$		MS^2[913]:<br>221.0659(100),<br>751.3987(12),<br>383.1182(10)	pratioside D1	+	+	+	+	+	+	+
139*	13.72	283.0612	283.0609	-1.0	$C_{16}H_{12}O_5$		MS^2[283]:<br>283.0610(100),<br>240.0421(20),<br>268.0375(10)	wogonin	-	-	-	-	-	+	+
140**	14.03	313.1081	313.1082	0.2	$C_{18}H_{18}O_5$		MS^2[313]:<br>207.0655(100),<br>295.2277(8)	6,8-dimethyl-4,'5,7-trihydroxyisoflavanones	+	+	+	+	+	+	+
141**	14.10	329.1031	329.1030	-0.2	$C_{18}H_{18}O_6$		MS^2[329]:<br>139.0389(100),	4',5,7-trihydroxy-8-methoxyhomois	+	+	+	+	+	+	+

							311.2231(10)	oflavones							
142***	14.22	429.2999	429.2987	-2.8	$C_{27}H_{40}O_4$	MS^2[429]:<br>411.2886(100),<br>393.2776(82),<br>229.1591(9)		Neoruscogenin isomer	+	+	+	+	+	+	+
143**	14.22	751.3910	751.3896	-1.9	$C_{39}H_{60}O_{14}$		MS^2[751]:<br>101.0231(100),<br>113.0231(38),<br>161.0446(28),<br>179.0553(15),<br>131.0337((7)	kingianoside A	+	+	+	+	+	+	+
144**	14.60	737.4107	737.4096	-1.4	$C_{39}H_{60}O_{13}$	MS^2[737]:<br>297.2211(100),<br>411.2893(83),<br>279.2109(69),<br>429.3003(55),<br>539.3332(10),<br>719.3937(9)		kingianoside B	-	-	-	-	+	+	+
145**	14.62	322.1079	322.1066	-4.0	$C_{19}H_{16}NO_4^+$	MS^2[322]:<br>307.0829(100),<br>279.0857(21)		Groenlandicine	-	-	-	+	+	+	+
146*	14.66	593.1301	593.1296	-0.7	$C_{30}H_{26}O_{13}$		MS^2[593]:<br>121.0284(100),<br>209.0452(58),<br>417.0984(7)	proanthocyanidin	+	+	+	+	+	+	+
147**	14.70	324.1236	324.1225	-3.4	$C_{19}H_{18}NO_4^+$	MS^2[324]:<br>292.0952(100),<br>309.0978(15),<br>280.0972(11)		Demethyleneberberine	-	-	-	-	+	+	+
148***	14.79	312.1241	312.1240	-0.5	$C_{18}H_{19}NO_4$		MS^2[312]:<br>297.1005(100),<br>191.0580(3)	N-trans-feruloyltyramine isomer	+	-	-	-	-	-	+
149**	15.04	279.2318	279.2313	-1.9	$C_{18}H_{30}O_2$	MS^2[279]:<br>81.0704(100),<br>95.0859(84),<br>109.1014(46),<br>261.2212(8),<br>243.2095(7)		α-linolenic acid	+	-	-	-	+	+	+
150***	15.05	737.4107	737.4100	-0.9	$C_{39}H_{60}O_{13}$	MS^2[737]:<br>297.2206(100),<br>411.2892(83),<br>279.2103(69),<br>429.3001(55),<br>539.3332(10),		kingianoside B isomer	-	-	-	-	+	+	+

						719.3937(9)									
151***	15.46	279.2318	279.2311	-2.6	$C_{18}H_{30}O_2$	MS^2[279]: 81.0703(100), 95.0857(81), 109.1012(46), 261.2212(8), 243.2095(7)		α-linolenic acid isomer	+	-	-	-	+	+	+
152***	15.90	279.2318	279.2312	-2.2	$C_{18}H_{30}O_2$	MS^2[279]: 95.0859(100), 109.1016(18), 81.0703(16), 261.2212(8), 243.2095(7)		α-linolenic acid isomer	+	-	-	-	+	+	+
153*	22.63	455.3531	455.3532	0.3	$C_{30}H_{48}O_3$		MS^2[455]: 408.9660(100), 255.2666(88), 412.1269(84)	betulinic acid	-	-	-	-	-	+	+

Refer to MSI standards, * denotes Level 1, ** denotes Level 2, *** denotes Level 3

Supplementary Table S2: Detailed information of the 37 standards in PCH

Name	Formula	Purity	Article number	Company
Hesperidin	$C_{28}H_{34}O_{15}$	HPLC＞98%	MUST-14081916	Chengdu Master Biotechnology Co., Ltd
Wogonin	$C_{16}H_{12}O_5$	HPLC＞98%	MUST190212-025	Chengdu Master Biotechnology Co., Ltd
Neochlorogenic acid	$C_{16}H_{18}O_9$	HPLC＞98%	MUST-15011412	Chengdu Master Biotechnology Co., Ltd
N-nornuciferine	$C_{18}H_{19}NO_2$	HPLC＞98%	220509	Chengdu Biochem Pure Biotechnology Co., Ltd
Quercetin	$C_{15}H_{10}O_7$	HPLC＞98%	C28J11Y116820	Shanghai Yuanye Biotechnology Co., Ltd
Riboflavin	$C_{17}H_{20}N_4O_6$	HPLC＞98%	J24F7R9932	Shanghai Yuanye Biotechnology Co., Ltd
Rutin	$C_{27}H_{30}O_{16}$	HPLC＞98%	AF8032520	Chengdu Efa Biotechnology Co., Ltd
Taxifolin	$C_{15}H_{12}O_7$	HPLC＞98%	AF22021002	Chengdu Efa Biotechnology Co., Ltd
L-tryptophan	$C_{11}H_{12}N_2O_2$	HPLC＞98%	AF20042702	Chengdu Efa Biotechnology Co., Ltd
Liquiritigenin	$C_{15}H_{12}O_4$	HPLC＞98%	AB0563-0020	Chengdu Efa

				Biotechnology Co., Ltd
Formononetin	$C_{16}H_{12}O_4$	HPLC＞98%	AB0816-0020	Chengdu Efa Biotechnology Co., Ltd
Roemerine	$C_{18}H_{17}NO_2$	HPLC＞98%	CFS202102	chemfaces
Isoferulic acid	$C_{10}H_{10}O_4$	HPLC＞98%	111698-201103	China Institute for Food and Drug Control
Hyperoside	$C_{21}H_{20}O_{12}$	HPLC＞98%	111521-201507	China Institute for Food and Drug Control
Luteolin	$C_{15}H_{10}O_6$	HPLC＞98%	M-007-190422	Chengdu Ruifensi Biotechnology Co., Ltd
Luteolin-7-O-glucoside	$C_{21}H_{20}O_{11}$	HPLC＞98%	M-025-181025	Chengdu Ruifensi Biotechnology Co., Ltd
Kaempferol	$C_{15}H_{10}O_6$	HPLC＞98%	S-014-171216	Chengdu Ruifensi Biotechnology Co., Ltd
Sinomenine	$C_{19}H_{23}NO_4$	HPLC＞98%	RFS-Q00711801013	Chengdu Ruifensi Biotechnology Co., Ltd
Chlorogenic acid	$C_{16}H_{18}O_9$	HPLC＞98%	L-007-171216	Chengdu Ruifensi Biotechnology Co., Ltd
Cryptochlorogenic acid	$C_{16}H_{18}O_9$	HPLC＞98%	Y-067-180425	Chengdu Ruifensi Biotechnology Co., Ltd
Naringenin	$C_{15}H_{12}O_5$	HPLC＞98%	Y-030-190812	Chengdu Ruifensi Biotechnology Co., Ltd
Salicylic acid	$C_7H_6O_3$	HPLC＞98%	VO0525000	Shandong West Asia Chemical Co., Ltd
Ferulic acid	$C_{10}H_{10}O_4$	HPLC＞98%	UD3365500	Shandong West Asia Chemical Co., Ltd
Azelaic acid	$C_9H_{16}O_4$	HPLC＞98%	CM1980000	Shandong West Asia Chemical Co., Ltd
Octanedioic acid	$C_8H_{14}O_4$	HPLC＞98%	20190620	Shandong West Asia Chemical Co., Ltd
3,4-dihydroxybenzoic acid	$C_7H_6O_4$	HPLC＞98%	UL0560000	Shandong West Asia Chemical Co., Ltd
Abscisic acid	$C_{15}H_{20}O_4$	HPLC＞98%	20190529	Shandong West Asia Chemical Co., Ltd
Proanthocyanidin	$C_{30}H_{26}O_{13}$	HPLC＞98%	wkq19050610	Sichuan Weikeqi Biotechnology Co., Ltd
Phlorizin	$C_{21}H_{24}O_{10}$	HPLC＞98%	wkq18050407	Sichuan Weikeqi Biotechnology Co., Ltd
P-Coumaric acid	$C_9H_8O_3$	HPLC＞98%	LotK1805057	aladdin
L-(-)-Malic acid	$C_4H_6O_5$	HPLC＞98%	M105695	aladdin
Caffeic acid	$C_9H_8O_4$	HPLC＞98%	C108306	aladdin
Kaempferol 3-glucorhamnoside	$C_{27}H_{30}O_{15}$	HPLC＞98%	HA020858	Herbest

Astragalin	$C_{21}H_{20}O_{11}$	HPLC＞98%	HA020488	Herbest
Apigenin-7-glucoside	$C_{21}H_{20}O_{10}$	HPLC＞98%	HA204891	Herbest
Nicotiflorin	$C_{27}H_{30}O_{15}$	HPLC＞98%	CFN99830	Wuhan Tianzhi Biotechnology Co., Ltd
Betulinic acid	$C_{30}H_{48}O_{3}$	HPLC＞98%	RDD-B01502009002	Chengdu Ruifenstedan Biotechnology Co., Ltd

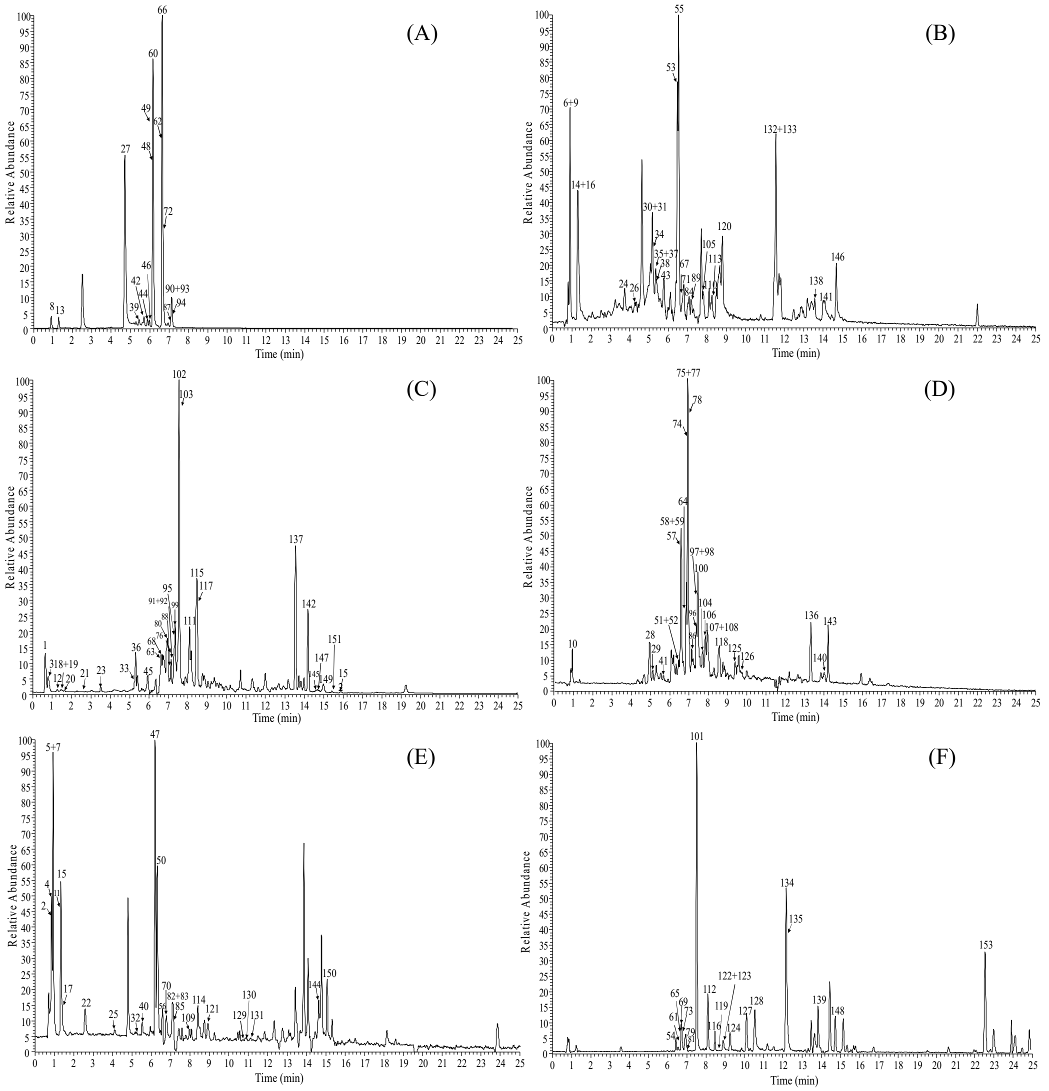

Supplementary Figure S1: The high-resolution extracted ion flow diagram of PCH. (A):Extract S in positive ion mode; (B):Extract S in negative ion mode; (C):Extract F in positive ion mode; (D):Extract F in negative ion mode; (E):Extract N in positive ion mode; (F):Extract N in negative ion mode. The Arabic numerals in the figure correspond to the compound numbers listed in Table 2S.
